# Seeing Is Believing: Visualizing Circular RNAs

**DOI:** 10.3390/ncrna6040045

**Published:** 2020-11-11

**Authors:** Pruthvi Raj Bejugam, Aniruddha Das, Amaresh Chandra Panda

**Affiliations:** 1 Institute of Life Sciences, Nalco Square, Bhubaneswar, Odisha 751023, India; raj.pruthvi@gmail.com (P.R.B.); ani.geneinvo@gmail.com (A.D.); 2 School of Biotechnology, KIIT University, Bhubaneswar, Odisha 751024, India

**Keywords:** circRNA, FISH, BaseScope, aptamer, localization, RNA-binding protein, miRNA

## Abstract

Advancement in the RNA sequencing techniques has discovered hundreds of thousands of circular RNAs (circRNAs) in humans. However, the physiological function of most of the identified circRNAs remains unexplored. Recent studies have established that spliceosomal machinery and RNA-binding proteins modulate circRNA biogenesis. Furthermore, circRNAs have been implicated in regulating crucial cellular processes by interacting with various proteins and microRNAs. However, there are several challenges in understanding the mechanism of circRNA biogenesis, transport, and their interaction with cellular factors to regulate cellular events because of their low abundance and sequence similarity with linear RNA. Addressing these challenges requires systematic studies that directly visualize the circRNAs in cells at single-molecule resolution along with the molecular regulators. In this review, we present the design, benefits, and weaknesses of RNA imaging techniques such as single-molecule RNA fluorescence in situ hybridization and BaseScope in fixed cells and fluorescent RNA aptamers in live-cell imaging of circRNAs. Furthermore, we propose the potential use of molecular beacons, multiply labeled tetravalent RNA imaging probes, and Cas-derived systems to visualize circRNAs.

## 1. Introduction

RNA molecules are conventionally known for the synthesis of proteins coded in the genome. However, this decade has seen an exploding number and type of RNA molecules in eukaryotic cells with the advent of next-generation sequencing. Interestingly, the noncoding (nc)RNAs contribute to more than 95% of the total RNA in the cell [1]. The ncRNA molecules such as snRNA, snoRNA, piRNA, tRNA, rRNA, and circular RNAs (circRNAs) work in coherence to express proteins from mRNAs. Over the past few years, next-generation sequencing technologies coupled with novel bioinformatic methods have led to the identification of ubiquitously expressed closed-loop circRNA molecules [2,3]. CircRNAs are widely expressed in all eukaryotes, conserved, and show cell type-specific expression [4]. Generally, the expression of the majority of circRNAs is less abundant than the counterpart linear RNAs [3]. Although some genes produce more than ten circRNAs, most genes with circular transcripts generate one or two circRNAs [5,6]. Based on the circRNA sequence overlap with the parental gene, circRNAs are categorized into various types, such as exonic circRNAs (ecircRNAs), exon–intron circRNAs (EIcircRNAs), circular intronic RNAs (ciRNAs), stable intronic sequence RNAs (sisRNAs), and tRNA intronic circular RNAs (tricRNAs) [7,8,9,10]. Circularization of exonic sequences and exon–intron sequences from the precursor RNAs through backsplicing generates ecircRNAs and EIcircRNAs, respectively [6,11]. Unlike linear RNAs, circRNAs are generated by the head-to-tail joining of circularizing exons through backsplicing (reviewed in [11]). Backsplicing of circRNAs from precursor mRNA requires the canonical spliceosomal machinery and the inverted intronic repeat sequences in the flanking introns of the circularizing exon [2,11,12]. Additionally, several RNA-binding proteins (RBPs), including MBNL1, NF90, quacking, and DHX9, have been shown to interact with the pre-mRNA and modulate circRNA biogenesis [11,12,13,14,15,16]. Furthermore, the intronic sequences have been shown to generate many lariat-derived ciRNAs and sisRNAs [9,10]. The ciRNAs escape the debranching process due to the C-rich 11 nt motif near the branch point and the GU-rich 7 nt sequence at the 5′ splice site [10]. TricRNAs are another class of circRNAs generated from the intron of the pre-tRNA. The bulge–helix–bulge motif of the pre-tRNA is spliced by the tRNA splicing endonuclease (TSEN) complex, followed by ligation of the intron ends by the RtcB to generate tricRNAs [8].

Hundreds of thousands of circRNAs have been identified in humans using high-throughput sequencing coupled with various bioinformatics tools (reviewed in [17]). However, the function of the majority of the circRNAs remains to be explored. Although much research has been performed to characterize the novel circular transcripts, no consensus has been reached to date on the biological function of these intriguing circles (reviewed in [7]). CircRNAs lack the 5′ cap and 3′ poly-A tails, which makes them more stable compared to linear RNA, making them the right candidate for gene regulation [3]. Increasing evidence suggests that circRNAs act as a decoy for RBPs, as a protein scaffold, as miRNA sponges, as a splicing regulator, and as a template for protein translation (reviewed in [18]). For example, ciRNAs and EIcircRNAs have been shown to interact with RNA pol II complex and U1 snRNA to regulate the transcription of the parental genes [10,19]. Additionally, backsplicing generating circMBL from exon 2 of the MBL gene competes with the pre-MBL mRNA splicing, leading to alternative splicing of MBL mRNA [13]. Since circRNA biogenesis leads to exon skipping, circRNA with the start codon of the parental mRNA can act as an mRNA trap and affect the protein expression from the parental mRNA [20]. In addition, circRNAs have been shown to contain miRNA target sites and act as competing endogenous RNAs (ceRNAs) for miRNAs [21]. miRNA sponging by circRNAs leads to increased expression of the cognate target mRNAs and has been extensively reviewed in [22]. Interestingly, miRNA association with circRNA has also been shown to regulate circRNA stability [23].

Increasing evidence suggests that circRNAs are aberrantly expressed in disease conditions and body fluids. Many recent studies have established that circRNAs may serve as a biomarker and therapeutic targets in various diseases, including cancer, diabetes, muscular atrophy, and aging (reviewed in [24,25,26,27]). CircRNAs are also increasingly shown to regulate various physiological and developmental processes by acting as a sponge for miRNAs or RBPs (reviewed in [24,26,27,28]). To understand the function of circRNA within a cell, it becomes essential to understand the expression pattern of circRNAs and their association with other biomolecules. Several computational tools have been developed to predict the association of circRNAs with cellular factors and predict their function (reviewed in [17]). In this review, we discuss different circRNA analysis methods with a particular focus on imaging techniques.

## 2. Methods to Analyze circRNAs

**Figure 1:**
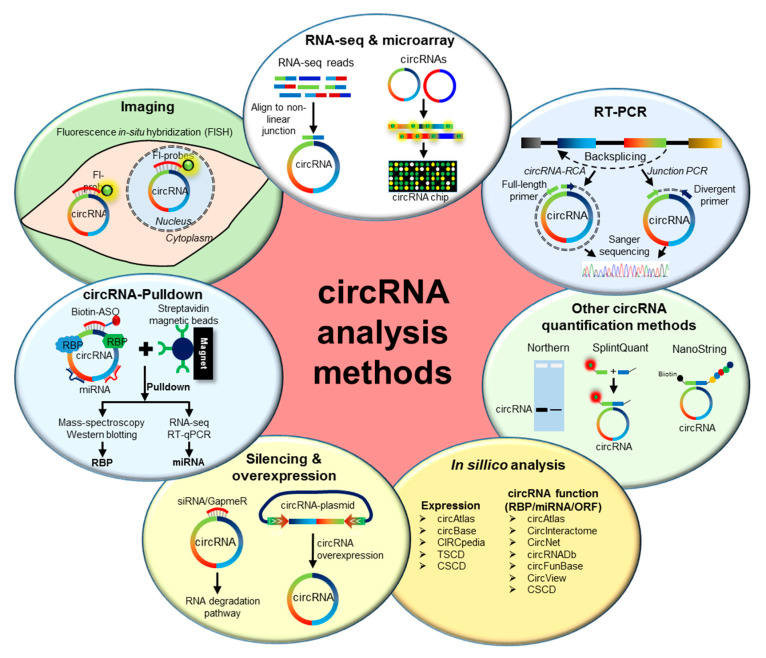
Schematic representation of different methods for circular RNA analysis. (From top clockwise) RNA-seq and circRNA microarrays are used for the genome-wide identification and quantification of circRNAs. RT-PCR of circRNA using the divergent and full-length primers across the backsplice junction, followed by Sanger sequencing, confirms the expression of specific circRNA. In addition, divergent primer PCR can be used for circRNA quantification. Other methods, such as northern blotting, SplintQuant, and NanoString can be used for the quantification of circRNAs. Several databases and web-tools have been developed for the in silico analysis of circRNA expression and function. Loss-of-function analysis for circRNA can also be performed using siRNA/GapmeR for circRNA silencing, while gain-of-function analysis can be achieved by overexpressing the circRNA of interest using a plasmid vector. The circRNA-associated cellular miRNAs and RNA-binding proteins (RBPs) can be analyzed by performing circRNA pulldown assays using antisense oligo-targeting circRNA junctions. Finally, circRNAs can be visualized in the cells using fluorescent-tagged probes targeting the backsplice junction of target circRNA.

Due to the lack of free ends and their variable size, circRNAs cannot be separated from other RNAs in gel and detected by molecular biology methods that rely on rapid amplification of cDNA ends (RACE) or RNA seq of poly-A enriched samples. Additionally, the circRNA sequence is the same as the parental mRNA, making it harder to detect and modulate their expression precisely. Intriguingly, the backsplice junction of circRNA provides a unique opportunity for their detection and manipulation. Several molecular biology approaches have been developed to identify and understand the molecular mechanism of circRNAs in cell physiology (Figure 1).

### 2.1. Genome-Wide Analysis of circRNA Expression

The last decade has seen significant advancements in genomic sequencing technologies, including ribosomal RNA depletion strategies, longer sequencing reads, greater sequencing depth, and novel algorithms to map the reads to the genome. These developments have led to the identification of splice variants of mRNAs, non-polyadenylated RNAs, including circRNAs. CircRNAs are identified from RNA-seq reads mapped to the same gene but in the opposite order compared to the linear genomic sequence [2,3,29]. However, the backsplice junction in RNA-seq can be formed not only because of backsplicing but with other mechanisms such as trans-splicing, tandem duplication, or by template switching of reverse transcriptase (reviewed in [29,30]). Various methods, such as poly-A RNA depletion, RNase R treatment, and RPAD (RNase R treatment, polyadenylation, and poly(A)+ RNA depletion), have been developed to enrich circRNA population for downstream analysis [30,31]. However, the complete removal of linear RNAs remains a challenge today. Several algorithms have been developed to identify backsplice junctions, full-length sequences, and quantify circRNAs in RNA-seq data (reviewed in [29]). However, there is no consensus on using the circRNA annotation algorithm to date due to the varying degree of overlap between different algorithms. In addition to RNA-seq, circRNA microarray platforms have been developed to identify and quantify known circRNAs [32]. Unlike RNA-seq, circRNA microarray uses probes targeting the backsplice junction of known circRNAs. Since the microarray probes have sequence similarity with linear RNAs, prior treatment of total RNA with RNase R may improve the specificity of detection. Unlike RNA-seq, circRNA microarray is limited to the number of circRNA probes present in the platform, cannot identify mature circRNA sequences, and cannot measure the ratio of circRNA to linear RNA.

### 2.2. Validation of circRNA Expression

Since both the high-throughput methods can identify false-positive circRNAs, other molecular methods like northern blotting, RT-PCR, and Sanger sequencing of PCR products are recommended for further validation and quantification of identified circRNAs. Due to the speed and convenience, PCR-based identification and quantification of circRNAs have been widely used. The divergent primers across backsplice junctions are used for PCR amplification of specific circRNAs [33]. Since divergent primers do not amplify linear RNAs, RNase R treatment of total RNA is not required for circRNA detection and quantification by PCR. The Sanger sequencing of the PCR product amplified with divergent primer can verify the backsplice junction sequence. However, divergent primer PCR cannot reveal the full-length sequence of circRNA and can amplify multiple circRNAs generated from the same region of the gene. The circRNA rolling circle amplification (circRNA-RCA) method was recently developed to amplify the full-length sequence of circRNA using primers on the junction sequence [34]. In addition, the splice variants of circRNA with the same backsplice junction can be identified with circRNA-RCA. However, this method cannot accurately quantitate different splice variants with the same circRNA junction. Although RT-PCR is the preferred method for circRNA identification and quantification, reverse transcription enzymes are known to interfere with circRNA quantification due to rolling circle amplification and template switching (reviewed in [29,30]). To overcome the RT-PCR bias, a few studies have used radioactive probes targeting the backsplice junction sequence for the northern blot analysis of circRNA [21]. Recently, other methods such as SplintQuant and NanoString have been developed for accurate quantification of endogenous circRNAs [35,36] (Figure 1).

### 2.3. Prediction of circRNA Expression and Function

Hundreds of thousands of circRNAs have been identified in human RNA-seq data using various circRNA annotation algorithms [17]. Several circRNA databases have been developed, containing various circRNA annotations, including tissue-specific expression, evolutionary conservation, disease association, circRNA–RBP interactions, and circRNA–miRNA interactions (Figure 1). Most databases contain human circRNAs, while some databases include monkey, mouse, rat, chicken, and yeast circRNAs. CircRNA databases such as TSCD, CSCD, CIRCpedia, circBase, and circAtlas include the expression levels of various circRNAs in different cells or tissues [37,38,39,40,41]. Several databases such as CSCD, circAtlas, circInteractome, circNet, starBase, and circFunBase have been developed to predict the function of circRNAs, including their interactions with miRNAs and RBPs [38,41,42,43,44,45]. A few databases also indicate their protein-coding potential, including circAtlas, circInteractome, circBank, and circRNADb [41,42,46,47]. Although all these databases predict the cellular function of circRNAs by interaction with cellular factors, they need to be validated experimentally.

### 2.4. Functional Characterization of circRNAs

The function of circRNA is studied with silencing or overexpression in the target cells or tissues. Given that backsplice junction is the unique sequence for circRNA, the siRNAs or GapmeRs are designed to target the junction sequence. GapmeRs and siRNAs targeting the junction can be manually designed and checked for the specific knockdown of target circRNA without affecting the counterpart linear RNA. Since siRNAs may act as miRNAs for the counterpart linear RNA due to similar sequences, GapmeR may be better for specific silencing of target circRNAs. Since backsplicing of circRNA is known to be enhanced by intronic inverted repeats, vectors have been developed with inverted repeat sequences where the circRNA of interest can be inserted for overexpression in the cells [48]. Since overexpression of circRNA from the vectors may generate a lot of linear RNAs with circRNA inserts and circular concatamers, the quantification of circRNA overexpression may be performed upon RNase R treatment [49]. For analyzing the molecular interaction of circRNA with predicted miRNAs or RBPs, circRNA pulldown assays can be performed using biotin-labeled antisense oligos targeting the junction sequence [50,51]. The pulldown of circRNA with streptavidin magnetic beads followed by RNA-seq or RT-qPCR and mass spectroscopy/western blot analysis can identify associated miRNAs and RBPs, respectively [50,51]. Additionally, immunoprecipitation of predicted RBPs of interest followed by RNA-seq, microarray, or RT-qPCR can identify circRNAs associated with specific RBPs (reviewed in [12]). Besides, fluorescence in situ hybridization (FISH) assays using probes specific for backsplice junction sequences have been used in many studies for studying the expression as well as localization of circRNAs in cells (Figure 1) [52,53]. In this review, we summarize different circRNA visualization tools and their use in analyzing circRNA expression and function.

## 3. CircRNA Detection and Quantification by RNA Imaging Techniques

They say “*seeing is believing*”. The ability to visualize a circRNA in situ not only confirms the existence of circRNA in a cell but also gives information about circRNA quantity, localization, and association with other biomolecules, which are critical to finding the function of circRNA in cell physiology. Interestingly, a recent study showed that the very well-studied oncogene circRNA *CDR1as* (*ciRS-7*) is not present in colon cancer cells, but abundantly expressed in stromal cells of the tumor microenvironment [54]. This study highlights the importance of circRNA visualization at the single-cell level for the accurate functional analysis of circRNAs [54]. Unlike linear mRNAs and lincRNA, circRNA detection with imaging techniques is challenging due to the lack of long stretches of unique sequences except for the circRNA junction. However, the probes targeting the circRNA junction offer a unique opportunity for circRNA imaging [52]. Given that circRNA function may vary depending on its presence in either cytoplasmic or nuclear compartments, it is crucial to check the localization of circRNAs in the cells. Localization in the cytoplasmic compartment would suggest a more miRNA- or RBP-sponging activity, whereas nuclear localization would suggest their potential regulatory role in transcription or splicing. In this review, we attempt to illustrate the current imaging approaches for circRNA analyses and discuss potential future developments in circRNA imaging.

Imaging techniques are broadly divided into the fixed-cell and live-cell imaging methods based on the kind of sample. The goal of both methods, however, is to visualize the target RNA and extract the relevant information. In a fixed-cell imaging approach, the cells are fixed to keep the cellular biomolecules intact. In contrast, the live-cell imaging approach usually tracks the dynamic movement of target RNAs over time.

### 3.1. Fixed-Cell circRNA Imaging

In situ hybridization (ISH) is one of the oldest imaging techniques used to detect the abundance and precise localization of RNAs in cells [55]. Traditionally, ISH is performed with radioactive probes that were expensive, time-consuming, and hazardous to human health. The utility of FISH increased in the late 1980s with the development of fluorescently labeled probes [56]. Since then, fluorescent probes are used in ISH that bind to nucleic acids based on sequence complementarity. Over time, even though the basic principle of this technique remains the same, there have been advances made in probe design, choice of the specimen, reduced hybridization times, and increased automation of the assay. Numerous variations in FISH methodologies have now been developed, which are much more sensitive, specific, and faster than the traditional FISH techniques. In recent years, there has been a significant advancement in the FISH techniques to reduce the off-target hybridization of the probes and enhance signal quality. Several approaches, such as FISH-STICS, padlock probes, and BaseScope techniques, have been developed to improve the signal quality. Since circRNA research is a relatively new field, only a few methods like single-molecule FISH (smFISH) and BaseScope have been used for the detection and localization of circRNAs.

#### 3.1.1. circRNA Imaging Using smFISH

**Figure 2:**
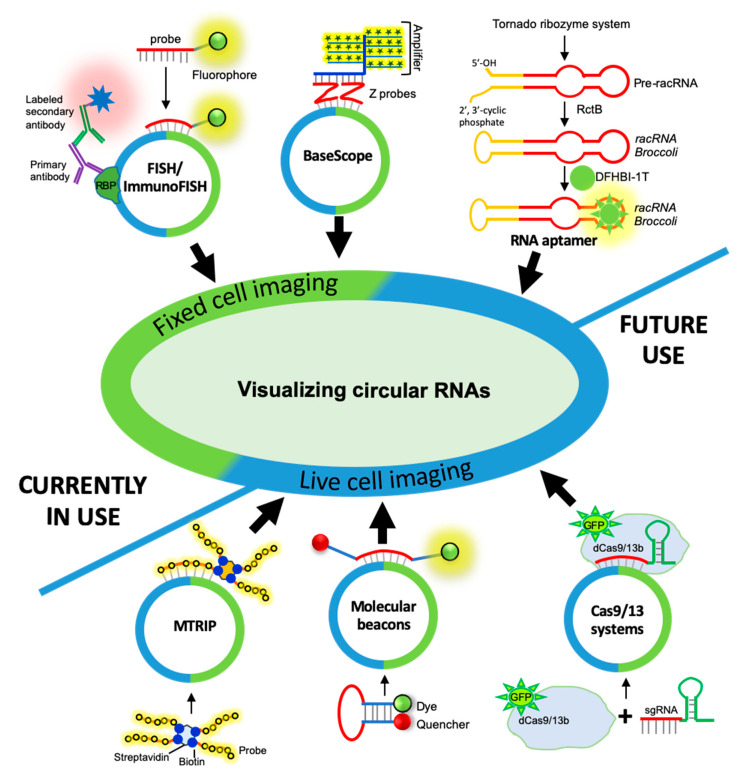
Schematic representation of different approaches for visualization of circular RNAs. (From top-left clockwise) circRNAs can be visualized in the fixed cells using single-molecule fluorescence in situ hybridization (smFISH) probes targeting the backsplice junction sequence. Additionally, proteins associated with the circRNA of interest can be co-detected using immunofluorescence assay while the circRNA can be detected with smFISH. CircRNAs may also be detected with the BaseScope Z pair probes targeting the backsplice junction, which are targeted by the pre-amplifier scaffold for fluorescent or chromogenic signal amplification. Fluorescent RNA aptamer sequences can be cloned into the circRNA sequence, which binds to the dye to emit fluorescence for live-cell visualization and tracking of circRNAs. The dCas imaging system requires the overexpression of GFP-tagged inactive Cas9/13b and sgRNA targeting the circRNA backsplice junction. The fully assembled dCas protein and sgRNA bound to target circRNA can be used for live-cell imaging of circRNAs. Molecular beacon probes targeting the circRNA junction sequence contain a fluorescent tag and a quencher. Upon binding to target circRNA in live cells, the dye dissociates from the quencher allowing visualization of target circRNA. The multiply labeled tetravalent RNA imaging probe (MTRIP) method uses internally labeled fluorescent probes targeting the circRNA junction, and the biotin tag at the end of the probe can tetramerize inside the cell with the streptavidin to amplify the signal for circRNA live-cell imaging.

The traditional FISH technique uses multiple short fluorescently labeled DNA probes that bind to the RNA. Due to the binding of multiple probes on a single RNA, the signal to noise ratio is enhanced. The image is then analyzed using appropriate fitting algorithms that detect spots in the image. Unlike traditional FISH, smFISH can detect even a single RNA molecule [57]. Since the circRNA junction is the unique sequence for a particular circRNA, the probes must target the backsplice junction of the circRNA of interest [52]. Several studies have used FISH or smFISH for quantification and localization of circRNAs (Figure 2, Table 1). The FISH assay using fluorescent probes targeting the junction of circRNA was successfully employed to detect the expression and localization of circRNAs, including CDR1as (ciRS-7), *circHECTD1*, *circARHGAP10*, *hsa_circRNA_103809*, *hsa_circ_0017639*, *circSAMD4A*, *circPVT1*, *circTTN*, *EIcircEIF3J*, *EIcircPAIP2*, *circRHOT1*, *circFAT1*, and *circTADA2A* [19,21,52,58,59,60,61,62,63,64,65,66,67,68]. Furthermore, recent studies employed FISH to confirm the cytoplasmic colocalization of circRNA and target miRNAs, including colocalization of miR-7 and *ciRS-7* in HEK293 and neuronal cells [21,52,69], miR-135a and ciRS-7 in bladder cancer [70], miR-330-5p and *circITCH* in cardiomyocytes [71], miR-143 and *circDLGAP4* in cerebral ischemia [72], *circCCDC9* and miR-6792-3p, *circRHOBTB3* and miR-654-3p in gastric cancer cells [73,74], *circFAM114A2* and miR-762 in urothelial carcinoma of the bladder [75], and circRNA *cZNF532* and miR-29a-3p in the cytoplasm of pericytes [76].

Additionally, ImmunoFISH is a variant of the smFISH technique where a combination of FISH and immunohistochemistry enables the detection of RNAs and proteins in the same sample. In ImmunoFISH, circRNA-associated protein is detected by immunofluorescence, while the target circRNA is detected with smFISH. ImmunoFISH confirmed the nucleolar colocalization of PA2G4 with *circERBB2* that regulates ribosomal DNA transcription in gallbladder cancer and the association of *circRHOT1* with TIP60 initiates NR2F6 expression in hepatocellular carcinoma [77,78]. Although FISH/smFISH is one of the most popular circRNA detection techniques, the visualization of low-abundance circRNA using a single probe targeting the circRNA junction might be challenging.

#### 3.1.2. circRNA Imaging Using BaseScope Assay

RNAscope technology is a recently developed version of the ISH technique that detects mRNAs and ncRNAs with a length of more than 300 nucleotides [90]. With its unique proprietary probe design that can amplify target-specific signals and not the background noise, RNAscope ensures a higher specificity as compared to the ISH techniques. It is also combined with immunohistochemistry to ensure the simultaneous detection of RNAs and proteins [91]. BaseScope assay is the advanced version of RNAscope, which is generally used for sequence detection of short target sequences of 50–300 bp (snoRNA, circRNA, miRNA, partially degraded RNA, and transiently expressed RNAs) using 1–3 Z pair probes. Since the circRNA junction sequence is the unique sequence, BaseScope can detect circRNA with a ZZ pair probe targeting the backsplice junction sequence (Figure 2). Hybridization of the signal pre-amplifiers using branched DNA technology and simultaneous signal amplification with chromogenic enzymes or florescent probes allows for the visualization of low-abundance circRNAs. Recently, the BaseScope assay was used in C2C12 muscle cells to visualize *circSamd4* that regulates myogenesis by sponging PUR proteins [51]. Similarly, several studies successfully employed a BaseScope assay for circRNA detection and localization in different cellular systems, including *circSHKBP1* in gastric cancer tissues [79], *circAR3* expression in prostate cancers [80], *circPLEKHM3* in the tumor samples [81], *circSlc45a4* in neuronal differentiation [82], *circCACNA2D1* and *circCACNA1E* in rhesus macaque brains [83], sisRNAs in HeLa and mouse 3T3 cells [84], and ciRS-7 in colon cancer and lesional skin [54,85]. Furthermore, a few studies used the BaseScope assay to detect circRNAs expressed during viral infection, including *circPANs* and *circK7.3s* in Kaposi’s sarcoma-associated herpesvirus (KSHV) replication and *circBHLF1* in Epstein Barr virus (EBV) infection [86,87].

### 3.2. Live-Cell Imaging of circRNAs

Fixed-cell imaging enables the observation of RNA only at a single time point in the cellular RNA pathway. It is generally useful for observing RNA localization. However, unlike this static observation, live-cell imaging adds a dynamic dimension that can track the entire RNA lifecycle. Live-cell RNA imaging captures many important events, such as RNA biosynthesis, splicing events, transport, function, and decay (reviewed in [92]). Unlike fixed-cell imaging, where cells become dead during fixation, live-cell imaging preserves the physiological condition of the cell. Tracking probes can either be endogenous or exogenous. Plasmid-based systems that express both the RNA and a fluorescent tag, such as the MS2-GFP system, are used that can bind specifically to the RNA of interest. Furthermore, multiple MS2-GFP binding domains have also been inserted in the RNA to enhance the signal intensity. Some other systems, such as PUM-HD, PP7, and Pepper, have also been developed to track endogenous mRNAs (reviewed in [92,93]). More recently, fluorescent proteins (GFPs) have been replaced by Halo and SNP tags, which can also be coupled with organic dyes (reviewed in [94]). Plasmid-derived probes (endogenous systems) offer flexibility; however, they do have limitations on the cell types that allow transfection of these systems and the copy numbers generated, possibly changing the fundamental stoichiometry of RNA expression. This led to the development of exogenous fluorophore-labeled antisense probes. Some of these are single-label probes (aptamers, molecular beacons) and some have multivalent labels (multiply labeled tetravalent RNA imaging probes (MTRIPs)). Although the RNA aptamer system has only been used for live-cell imaging of circRNAs, we propose the potential uses of other live-cell approaches that can be used for circRNA imaging.

#### 3.2.1. Fluorescent RNA Aptamers

RNA aptamer-based imaging systems require an aptamer sequence fused with the transcript of interest that can only be visualized when the dye is captured by the aptamer structure with no background issue during RNA tracking experiments (reviewed in [92]). The first small molecule-based fluorophore-binding aptamer (Spinach) system mimicked the structure of GFP. Since then, other aptamers such as Broccoli and Spinach 2 have been developed. More recently, aptamer dye systems such as Broccoli-DFHB1, and Corn-DFHO have been developed, showing higher thermal stability and improved photostability (reviewed in [92]). One potential limitation of these aptamers is the presence of G-quadruplexes that is often critical for fluorophore binding and rigidification of the aptamer. Newer aptamer-based systems, such as SRB-2, RhoBAST, and Gemini-561, do not have this structure and have been shown to have increased photostability (reviewed in [92,93]). In contrast to these aptamer-based systems, aptamer systems inspired by naturally occurring riboswitches have also been developed. Riboglow has been developed, which makes use of the covalent conjugation of cobalamine, a metabolite that binds to the riboswitches. The binding of cobalamine to an RNA riboswitch results in the separation of the quencher and fluorophore than can turn on the fluorescence for visualization (reviewed in [93]).

Although several aptamer–dye systems have been developed to visualize linear RNAs in live cells, only a few studies have used RNA aptamers for visualizing circRNAs. Given the tRNA introns are spliced to form RNA circles known as tricRNA, a recent study engineered aptamers containing circRNAs by inserting sequences corresponding to Broccoli and Spinach 2 RNA aptamers in the tRNA intron [8,88]. Interestingly, the chimeric circRNA with these aptamers bind to the chromophores, like DFHBI-1T, resulting in green fluorescence that mimics GFP with very low background fluorescence [88]. Furthermore, an artificial autocatalytic tornado system was developed for the overexpression of ribozyme-assisted circRNA (racRNA) with Broccoli aptamers and visualized in live cells using DFHBI-1T (Figure 2) [89].

#### 3.2.2. Cas-Derived Fluorescent Protein

Although the CRISPR/Cas system is well known for gene silencing and genome editing, fluorescently labeled modified Cas proteins have recently been used for live-cell tracking of target RNA with high specificity (reviewed in [95]). Several systems, such as dCas13, dCas9, dPspCas13b, have recently been developed for real-time RNA imaging where the Cas protein homologs are fused with fluorescent proteins such as EGFP [95,96,97]. With a discrimination ability of even a single base mismatch, CRISPR/Cas technology can detect RNAs with very high specificity. These CRISPR-based systems are compatible with both the fluorescent-based and the chromogenic method of detection. Although the CRISPR/Cas system is widely used for gene silencing and editing, a recent study used the dCas9-mCherry-based system to image the GAPDH mRNA in live cells [97]. Furthermore, dLwaCas13a and dCas13d systems have been used for real-time imaging of the RNA in living cells [96]. We hypothesize that single-guide RNA (sgRNA) targeting the backsplice junction sequence coupled with the fluorescent-labeled dCas9/dCas13d may be used for live-cell imaging of circRNAs (Figure 2).

#### 3.2.3. Molecular Beacons

The molecular beacon (MB) was first developed in 1996 for nucleic acid amplification assays as well as RNA tracking in live cells [98]. Unlike fluorescent probes used in FISH, molecular beacons use a probe complementary to target RNA labeled with a fluorophore and a quencher [99]. Molecular beacons form a stem–loop structure in the native state where the fluorophore is quenched by the quencher, giving no fluorescence. Upon hybridization to the target RNA, the probes become fluorescent as the quencher and the fluorescent dye move apart due to the conformational reorganization. Molecular beacons are promising probes for real-time monitoring of RNA dynamics within living cells and tissues (reviewed in [100]). Although MBs have not yet been used for circRNA imaging, we believe that the MB probe targeting the backsplice junction sequence of circRNA may enable live cell tracking of circRNAs of interest (Figure 2).

#### 3.2.4. Multiply Labeled Tetravalent RNA Imaging Probes

Multiply labeled tetravalent RNA imaging probes (MTRIPs) can be used for RNA imaging with high specificity. To increase the sensitivity of nucleic acids based on exogenous probes, Santangelo et al. developed MTRIPs by conjugating two to four fluorophores to a single nucleic acid. These nucleic acids (also referred to as ligands) were combined using streptavidin such that the probe brightness was increased four fold [101,102]. One key advantage of using MTRIPs is that multiple probes with multiple fluorophores per target RNA achieve several-fold higher brightness as compared to the traditional method of using fluorescent protein-based RNA detection. We hypothesize that MTRIPs targeting the circRNA junction could help in specific detection and live-cell imaging of circRNA (Figure 2).

## 4. Limitations and Additional Considerations for circRNA Imaging Techniques

CircRNAs regulate cell physiology by acting as sponges for microRNAs, a decoy for RBPs, competitors of splicing regulators, and substrates for protein translation (reviewed in [18]). Although circRNAs are believed to be localized into the cytoplasm and regulate the function of RBPs and miRNAs, several circRNAs have been reported to localize into the nucleus to regulate nuclear events, including transcription and RNA splicing [10,19,69]. We have discussed recent imaging techniques for identification, quantification, and localization of circRNA in the cells (Figure 2, Table 1). All these methods for visualization of circRNAs have their strengths and weaknesses, which need to be considered during experimental design (Table 2).

Given that circRNAs are generated from pre-mRNAs, the body of the circRNAs resembles the counterpart linear RNAs, limiting the specific probe to be complementary only to the junction sequence of circRNA. As discussed above, smFISH employs a fluorescent-labeled probe that is complementary to the circRNA junction sequence and useful for detecting abundant circRNAs (Table 1). It might be challenging to detect low-abundance circRNAs with smFISH. Since most circRNAs have low abundance, with a few copies per cell, the BaseScope assay is very useful in circRNA imaging. The smFISH and BaseScope technique for circRNA imaging can also be multiplexed to detect multiple target RNAs and proteins using probes/antibodies labeled with different fluorophores or chromophores in a single assay. However, both smFISH and BaseScope assays are limited to fixed cells only.

Live-cell imaging of circRNAs using RNA aptamers has only been reported recently, utilizing the tricRNAs and racRNAs [8,88,89]. This system holds promise for visualizing circRNAs with other RNAs and labeled proteins. However, additional research is required to determine the size of the circRNA that can be generated with the RNA aptamers using the tRNA or ribozyme-assisted splicing machinery. Additionally, the insertion of RNA aptamer sequences into the circRNA of interest may alter the aptamer folding and perturb circRNA interaction with RBPs and miRNAs, depending on the surrounding sequences, temperature, and cellular salt concentration. Furthermore, the overexpression of aptamer-containing circRNA in the cells may leave some unprocessed linear RNA with the aptamer sequence, which could interfere with circRNA tracking.

Furthermore, the inactive Cas-13 system with a fluorescent tag and the sgRNA targeting the circRNA junction could help live-cell tracking of circRNAs [96,97]. However, the gRNA specificity and the localization of the Cas protein needs to be verified for accurately tracking the target circRNA. Molecular beacon probes conjugated with a fluorophore and quencher could target the junction sequence of circRNA [99,100]. They can be introduced into cells where binding of the probe to the circRNA separates the fluorophore and quencher, allowing the visualization of the circRNA in real time. Like smFISH, this assay could be useful for the visualization of abundant circRNAs in fixed cells and live cells. The MTRIP method discussed here could use a multiply labeled fluorescent probe against the circRNA junction, and the streptavidin core can be introduced into the cell for live-cell imaging of target circRNA [101,102]. Finally, the success of smFISH, BaseScope, molecular beacons, the Cas-13 system, and MTRIP assays depends on the accessibility of the circRNA junction sequence, which might be hidden or bound with RBPs, preventing the probe binding.

## 5. Conclusions and Future Perspectives

Recent advancements established that circRNAs are stable, ubiquitously expressed, abundant, and evolutionarily conserved RNA families (reviewed in [7]). There is rising recognition that circRNAs regulate critical cellular processes, including transcription, mRNA splicing, mRNA stability, and translation, by associating with cellular molecules like proteins, miRNAs, and snRNAs (reviewed in [7,18]). Many circRNAs are dysregulated in diseases and are secreted into body fluids, serving as a diagnostic biomarker (reviewed in [28,103]). Insight into the circRNA life cycle from biogenesis by backsplicing, through transport into the cytoplasm or extracellular space, and to the regulatory function of sponging miRNA/RBPs have been studied extensively using molecular biology techniques. Although RNA imaging techniques for mRNAs and lncRNAs have been extensively used and have evolved to understand the molecular dynamics, visualization of circRNA is challenging due to their low abundance, and only one probe could target the circRNA junction sequence. While RNA imaging techniques such as smFISH and BaseScope have been used in the last couple of years to detect circRNAs in fixed cells, the visualization of circRNA in live cells is in its infancy. The in-depth understanding of circRNA-related cellular events would benefit from future circRNA live-cell imaging assays.

As discussed in this review, the spatiotemporal interaction of circRNAs with cellular machinery in live cells must be investigated in the context of (1) transcription of pre-mRNA and associated splicing machinery during circRNA biogenesis, which may change with the cellular state, stimuli, transcription speed, and disease conditions; (2) circRNA localization into different subcellular compartments (nucleus, cytoplasm, mitochondria, endoplasmic reticulum); (3) circRNA trafficking from nucleus to cytoplasm and packaging into exosomes for secretion; (4) the dynamic interaction of circRNA with RBPs and miRNAs affecting cellular processes. Given that immense research is underway to elucidate circRNA function, future studies must include these considerations to improve understanding of circRNA dynamics in human physiology and pathology.

## Figures and Tables

**Table 1:** List of circRNAs analyzed by imaging techniques.

	CircRNA Name	Cell or Tissue	Purpose	Reference
**FISH**	***circHECTD1***	Ischemic brain tissues	Quantification and localization	[58]
	***circARHGAP10***	Human non-small cell lung cancer tissues	Quantification and localization	[59]
	***hsa_circRNA_103809***	Hepatocellular carcinoma tissues		[60]
	***hsa_circ_0017639***	Gastric cancer cells	Localization	[61]
	***circSAMD4A***	Preadipocytes	Localization	[62]
	***circPVT1***	Human non-small cell lung cancer tissues	Localization	[63]
	***circTTN***	Bovine primary myoblasts	Localization	[64]
	***circRHOT1***	PANC-1 and Capan-2 pancreatic cancer cells	Localization	[65]
	***circEIF3J & circPAIP2***	HEK293 cells	Localization	[19]
	***CDR1as (ciRS-7)***	Adult brain, bladder cancer, and HEK293 cells	Localization	[21,66,69,70]
	***CircFAT1***	HOS and 143B osteosarcoma cells	Localization	[67]
	***circTADA2A***	HOS and 143B osteosarcoma cells	Localization	[68]
	***circITCH***	Cardiomyocytes (hiPSC-CMs)	Localization	[71]
	***circDLGAP4***	Brain endothelial cells	Quantification and localization	[72]
	***circRHOBTB3***	HGC27 and AGS cells	Localization	[73]
	***circCCDC9***	MKN45 and AGS cells	Localization	[74]
	***circFAM114A2***	UCB cells	Localization	[75]
	***circZNF532***	Pericytes	Localization	[76]
	***circERBB2***	GBC-SD cells, SGC-996 cells	Localization	[77]
	***circRHOT1***	Hepatocellular carcinoma (HCC)	Localization	[78]
**BaseScope**	***circSamd4***	C2C12 myoblasts	Localization	[51]
	***circSHKBP1***	HGC27 cells	Localization	[79]
	***circAR3***	PCa tumor samples	Localization	[80]
	***circPLEKHM3***	A2780 and OV90 cells	Localization	[81]
	***circSlc45a4***	E15.5 mouse cortices	Localization	[82]
	***circCACNA2D1* and *circCACNA1E***	Rhesus macaque brain	Localization	[83]
	***sisRNAs***	HeLa and mouse 3T3 cells	Localization	[84]
	***CDR1as (ciRS-7)***	Colon cancer and lesional skin	Quantification	[54,85]
	***circPANs* and *circK7.3s***	Kaposi’s sarcoma-associated herpesvirus (KSHV) infected tumor	Quantification	[86]
	***circBHLF1***	Epstein Barr virus (EBV)	Localization	[87]
**Aptamer**	**tricRNA: Broccoli** **tricRNA: Spinach2**	HEK293T cells	Live cell tracking	[88]
	***tricY*: Broccoli** ***racRNA*: Broccoli**	HEK293T, HepG2, HeLa, and COS-7 cells	Live cell tracking	[89]

**Table 2:** Overview of various circRNA imaging techniques.

CircRNA Imaging Method	*smFISH and ImmunoFISH*	*BaseScope*	*RNA Aptamer*	*CRISPR-Cas System*	*Molecular Beacon*	*MTRIP*
**Mechanism**	Single fluorescent-labeled antisense probe targeting the backsplice junction of circRNA-associated protein detected with fluorescent antibodies.	One ZZ probe pair targets the circRNA junction.	A short stretch of RNA sequence introduced to target circRNA binds to fluorochrome for live-cell imaging.	SgRNA-mediated specific detection of target RNA by the fluorescent protein-tagged Cas protein.	Hairpin-shaped molecules with an internally quenched fluorophore whose fluorescence is restored when they bind to a target RNA.	Multiply labeled tetravalent RNA imaging probe that identifies RNA, enhanced signal to background ratio.
**Advantages**	Probes are inexpensive, easy to synthesize, and easily penetrate the cells. Multiplexing with other circRNAs, miRNAs, or target proteins.	Very sensitive, allows detection of single-copy circRNAs.	Thermally stable, robust in binding to dye. Cost-effective and low background. Suitable for live-cell imaging.	Live-cell imaging. Very sensitive and specific for target RNA.	Live-cell imaging. Low signal-to-background fluorescence from unbound dye. Capable of multiplexing.	Live-cell imaging. Higher specificity and high signal intensity.
**Limitations**	Time-consuming and works only in fixed cells. Difficult to visualize low-abundance circRNAs. CircRNA probes may target the parent mRNA due to sequence similarity.	Expensive and not suitable for live-cell imaging.	Limited knowledge on the optimal placing of the aptamer within circRNA. Limited availability of fluorophores in aptamer dye systems. Fluorophores can sometimes be cytotoxic.	Limited resources available for designing specific sgRNA. It cannot multiplex.	Requires extensive technical optimization of probe design and hybridization technique. Introduction into the cell may be challenging.	Expensive and difficult to synthesize. Introduction into the cell may be challenging.
